# Pharmacotherapies for Drug-Induced Liver Injury: A Current Literature Review

**DOI:** 10.3389/fphar.2021.806249

**Published:** 2022-01-05

**Authors:** Meng Li, Qiong Luo, Yanyan Tao, Xin Sun, Chenghai Liu

**Affiliations:** ^1^ Institute of Liver Diseases, Shuguang Hospital Affiliated to Shanghai University of Traditional Chinese Medicine, Shanghai, China; ^2^ Shanghai Key Laboratory of Traditional Chinese Clinical Medicine, Shanghai, China; ^3^ Key Laboratory of Liver and Kidney Diseases, Ministry of Education, Shanghai, China; ^4^ Shanghai Innovation Center of TCM Health Service, Shanghai, China

**Keywords:** drug-induced liver injury, acute liver failure, pharmacotherapy, hepatoprotective drug, anticholestatic drug

## Abstract

Drug-induced liver injury (DILI) has become a serious public health problem. For the management of DILI, discontinuation of suspicious drug or medicine is the first step, but the treatments including drugs and supporting approaches are needed. Reference to clinical patterns and disease severity grades of DILI, the treatment drugs were considered to summarize into hepatoprotective drugs (N-acetylcysteine and Glutathione, Glycyrrhizin acid preparation, Polyene phosphatidylcholine, Bicyclol, Silymarin), anticholestatic drug (Ursodeoxycholic acid, S-adenosylmethionine, Cholestyramine), immunosuppressants (Glucocorticoids) and specific treatment agents (L-carnitine, Anticoagulants). The current article reviewed the accumulated literature with evidence-based medicine researches for DILI in clinical practice. Also the drawbacks of the clinical studies involved in the article, unmet needs and prospective development for DILI therapy were discussed.

## Introduction

With the extensive use of drugs and the continuous development and application of new drugs, the safety of drug use is widely concerned. Drug-induced liver injury (DILI) is one of the most common and serious adverse drug reactions (ADRs) (Miguel et al., 2012). DILI can be caused by various prescription or non-prescription chemical drugs, including biological agents, traditional Chinese medicine (TCM), natural medicine (NM), health care products (HP), dietary supplements (DS), their metabolites and excipients (European Association for the Study of the Liver. 2019). To date, with no specific diagnostic markers, diagnosis of DILI has been very challenging, and the pathogenesis has not been understood completely. Misdiagnosis and inappropriate treatment are quite likely resulting in unavoidable systematic errors in the efficacy of DILI interventions. In clinical practice, Roussel Uclaf Causality Assessment Method (RUCAM) and liver biopsy could help clinicians to better diagnose and differentiate DILI. Most countries and regions in the world also have summarized the empirical therapeutic strategies for DILI and formed guidelines. Actually, DILI could be categorized three clinical patterns as hepatocellular, cholestatic or mixed injury (liver damaged target cells) based on serum ALT and ALP ratio from the first available biochemical test (Chalasani Naga et al., 2021). So, the therapeutic strategies of DILI, especially therapeutic drugs, can be also classified reference to these three clinical patterns of DILI. In the article, the therapeutic agents of DILI were reviewed and summarized based on the published literature. Our search was performed in PubMed, CNKI and Web of Science, searching for the terms “drug-induced liver injury,” “acute liver failure,” “treatment,” “pharmacotherapy,” “protect hepatocyte,” “cholagogue” and/or “trial.” Clinical studies, case reports, reviews and guidelines were included. However, the published data about therapeutic drugs of DILI were heterogeneous. More well-designed studies, including control trials, are needed and a multicentre approach may be required in the future.

## Basal Principles of DILI Management

The most definitive treatment for DILI is to suspend the offending drug(s) and to avoid re-exposure (European Association for the Study of the Liver. 2019; Chalasani Naga et al., 2021). After drug withdrawal, most liver damage can recover spontaneously, with recovery time ranging from a few days to a few weeks (Devarbhavi et al., 2010). Actually, spontaneous recovery after discontinuation of the susceptible drugs or medicines is important criterion in the causality assessment. However, multiple suspicious drugs are often used together in clinical practice, it is difficult to identify which drugs or drug interactions cause liver injury. Moreover, many drugs are necessary for treatment of primary disease, and discontinuation of drugs may lead to serious consequences, which is also a major difficulty in the clinical treatment of drug-induced liver injury. Besides, small elevations in liver parameters induced by drugs may be transient in nature and may revert to baseline even when therapy is continued (such as antituberculosis drug and statin). These elevations may represent mild liver injury with spontaneous resolution or “adaptation.” So far, the mechanisms potentially underlying this “adaptation phenomena” were not known (Watkins Paul, 2005; Aithal et al., 2011). Therefore, it is necessary to fully evaluate the risk of primary disease progression caused by drug withdrawal and exacerbation of liver injury caused by continued drug use. To avoid unnecessary drug withdrawal, the International Serious Adverse Events Consortium (iSAEC) recommended in 2011 the modified biochemical criteria for the identification of DILI as reaching any of the following items: 1) ALT ≥5×upper limit of normal (ULN); 2) ALP≥2 ULN, especially in patients with elevated 5′-nucleotidase or GGT, and without bone-diseases-related ALP elevation; 3) ALT ≥3 ULN and TBil ≥2 ULN (Aithal et al., 2011). Although not clinical diagnostic criterion for DILI, it can provide a reference for treatment decision-making. Besides, the DILI patient should be educated and given alert card to avoid repeating the use of the offending agent or similar ones with known cross reactivity because continued exposure is associated with the development of severe or chronic liver diseases (Aithal and Day, 1999). The pharmacotherapy of DILI is particularly important, especially for patients with severe hepatitis and acute liver failure (ALF). Pharmacotherapy strategies are mainly aimed at protecting hepatocytes, scavenging free radicals and anti-oxidation, stabilizing hepatocytes membrane, detoxifying, reducing transaminase, immune regulation and so on. As we mentioned, it requires appropriate drug administration according to the clinical pattern of DILI. This part will be summarized detailedly in the following sections. In addition, patients with ALF or subacute liver failure (SALF) should consider artificial liver supporting treatment and liver transplantation (Devarbhavi et al., 2021).

Shown as Table 1, the basic principles for treating DILI are 1) identifying the offending drug(s) and prompt withdrawal before irreversible liver damage; 2) monitoring liver biochemistries, assessing risk and benefit of dubious drug(s) for primary diseases; 3) appropriate medication is selected reference to the clinical pattern of DILI; 4) artificial liver supporting therapy and liver transplantation for ALF/SALF patients.

**TABLE 1:** Basal principles of DILI management.

Principles	Describes	
I. Identify & Withdrawal	Identifying the offending drug(s) and prompt withdrawal before irreversible liver damage	
II. Monitor & Assessment	Monitoring liver biochemistries, assessing risk and benefit of dubious drug(s) for primary diseases	
III. Appropriate medication	Hepatocellular type	Give priority to with hepatoprotective drugs (eg. NAC, MgIG, PPC, etc.)
(according to the clinical pattern of DILI)	Cholestatic type	Give priority to with anticholestatic drugs (eg. UDCA, SAMe, etc.)
		Immunosuppressants (eg. GCs)
	Specific type	Specific treatment agents (eg. LC, Anticoagulants, etc.)
IV. Liver transplantation	Artificial liver supporting therapy and liver transplantation for ALF/SALF patients	

Note: NAC, N-acetylcysteine; MgIG, magnesium isoglycyrrhizinate; PPC, Polyene phosphatidylcholine; UDCA, Ursodeoxycholic acid; SAMe, S-adenosylmethionine; GCs, Glucocorticoids; LC, L-carnitine; ALF, acute liver failure; SALF, subacute liver failure.

## Pharmacotherapy Strategies

It required appropriate pharmacotherapy to reduce liver damage after stopping use the offending drug(s), when serum transaminase of patients could not normalize and bilirubin remained abnormal within a long time. Reference to the clinical patterns of DILI, pharmacotherapy was considered to classify as follows: 1) hepatoprotective drugs, 2) anticholestatic drug, 3) immunosuppressants, 4) specific treatment. And their efficacy for DILI based on clinical practice evidence was summarized in Table 2.

**TABLE 2:** Pharmacotherapy strategies of DILI.

Drugs Classification	Drugs name	Author & Year	Type of Study	Culprit drug	Outcomes/Conclusion
Hepatoprotective Drugs	NAC and GSH	Prescott et al. (1979)	Comparative Study	Paracetamol	LFP, SVI
		Moosa et al. (2020)	Randomized, double-blind, placebo-controlled trial	Anti-tuberculosis drug	SHS
		Borlak et al. (2018)	Retrospective cohort study	Flupirtine	LFP
		Patel et al. (2019)	Case Reports	Tyrosine Kinase Inhibitor	LFP
		Elliott et al. (2016)	Case Reports	Norfloxacin	LFP
		Lee et al. (2009)	Randomized Controlled Trial	Non-APAP agents	LFP, SVI
		Reuben et al. (2010)	Prospective observational study	Multiple drugs	LFP, SVI
		Squires et al. (2013)	Randomized Controlled Trial	Non-APAP agents	NSD
	Glycyrrhizin acid preparation	Wang et al. (2019)	Randomized, double-blind, controlled trial	Multiple drugs	LFP
		Tang et al. (2012)	Phase Ⅲ clinical trial	Anti-neoplastic drugs	LFP
	Bicyclol	Naiqiong et al. (2017)	Randomized Controlled Trial	Statin	LFP
		Chen et al. (2018)	Comparative Study	Anti-tuberculosis drug	LFP
		Li et al. (2018)	Retrospective cohort study	Anti-neuropathy drug	LFP
		Zhou et al. (2015)	Randomized controlled study	Anti-psoriasis drug	LFP
	Polyene phosphatidylcholine	Lei et al. (2021)	Literature analysis	Multiple drugs	LFP
		Liu et al. (2021)	Retrospective controlled study	Anti-tuberculosis drug	LFP
	Silymarin	Enjalbert et al. (2002)	Retrospective analysis	Toadstool	SVI
		Grabhorn et al. (2013)	Retrospective controlled study	Toadstool	SVI
		Masoumeh et al. (2015)	Open-label, randomized clinical trial	Anti-convulsive drugs	LFP
		Majid. et al. (2019)	Randomized, double-blind clinical trial	Anti-tuberculosis drug	NSD
		Zhao et al. (2021)	Retrospective cohort study	Multiple drugs	LFP
Anticholestatic drugs	Ursodeoxycholic acid	Lang et al. (2019)	Prospective pilot study	Anti-mycobacterial infections drugs	LFP
		Sun et al. (2015)	Retrospective controlled study	Multiple drugs	LFP
		Asgarshirazi et al. (2015)	Prospective observational study	Anticonvulsants	LFP
		Saito et al. (2016)	Retrospective controlled study	Anti-tuberculosis drug	NSD
		Bessone et al. (2021)	Review	Multiple drugs	UK
		Robles-Díaz Mercedes. et al. (2021)	Systematic review	Multiple drugs	UK
	S-adenosylmethionine	Almasio et al. (1990)	Review	Multiple drugs	LFP
		Perlamutrov et al. (2014)	Prospective observational study	Immune-suppressive therapy	LFP
		Hayashi and Fontana. (2014)	Prospective observational study	Chemotherapy	LFP
		Santini et al. (2003)	Prospective observational study	Chemotherapy	LFP
	Cholestyramine	Stine and Lewis. (2016)	Review	Leflunomide	UK
Immunosuppressants	Glucocorticoids	Hou et al. (2012)	Retrospective cohort study	Multiple drugs	LFP, SHS
		Hu et al. (2016)	Retrospective cohort study	Multiple drugs	LFP, SHS, SVI
		Sundaram et al. (2020)	Case Reports	Imatinib, cephalexin	LFP
		Herrero-Herrero and García-Aparicio. (2010)	Case Reports	Amoxicillin-clavulanate	LFP
		Karkhanis et al. (2014)	Retrospective cohort study	Multiple drugs	NSD
		Wan et al. (2019)	Prospective observational study	Multiple drugs	NSD
		Pang et al. (2018)	Retrospective controlled study	Multiple drugs	NSD
		Hu and Xie. (2019)	Review	Multiple drugs	UK
Specific drugs treatment	L-carnitine	Bohan et al. (2001)	Retrospective cohort study	Valproic acid	LFP
	Anticoagulants	Wang et al. (2016)	Retrospective cohort study	Gynura segetum	SVI
		Richardson et al. (1998)	Retrospective observational study	HSCT	LFP, SVI
		Richardson et al. (2016)	historically controlled, multicenter, open-label, phase iii trial	HSCT	LFP, SVI

Note: LFP, Liver function parameters improvement (including ALT, AST, ALP, GGT, TBIL, INR, or PT, etc.); SHS, Shorter hospital stay; SVI, Survival improvement (including the extension of survival time or improved survival rates); NSD, No significant difference; UK, Unknown or remain controversial.

### Hepatoprotective Drugs

Hepatoprotective drugs refer to drugs that can improve liver function, promote liver cell regeneration and/or enhance liver detoxification, and there is no unified understanding in regard to its classification. According to the different mechanism of action, it can be roughly divided into detoxification drugs (eg. NAC and GSH), anti-inflammatory drugs (eg. Glycyrrhizic acid preparation), hepatocyte membrane protector (eg. PPC), antioxidant drugs (eg. Bicyclol, Silymarin) (Chinese Society of Infectious Diseases, 2014).

#### N-Acetylcysteine and Glutathione

NAC is an N-acetylated derivative of L-cysteine (L-Cys) and a precursor of Glutathione (GSH), and has the pharmacological effects of anti-oxidation, anti-inflammation, dilatation of microvessels and protection of DNA molecules (Dekhuijzen, 2004). It is the only antidote approved by the US Food and Drug Administration (FDA) in 2004 for acetaminophen (APAP)-induced DILI. In an observational study from Edinburgh, intravenous NAC was claimed to be equally as effective as cysteamine and methionine and free of adverse effects (Prescott et al., 1979). Ever since, NAC has been accepted as an antidote for acetaminophen (APAP) overdose either intravenously or orally (Chiew et al., 2018). In addition, NAC can also be applied to liver injury caused by other drugs and DILI with ALF. Moosa MS et al. conducted a randomized, double-blind, placebo-controlled trial to assess whether intravenous NAC hastens liver recovery in hospitalized adult patients with anti-tuberculosis (Anti-TB) DILI. The results revealed that NAC did not shorten time to ALT<100 U/L in participants with Anti-TB DILI, but significantly reduced length of hospital stay (Moosa et al., 2020). In another study NAC together with prednisolone when used in cases of severe idiosyncratic DILI due to fupirtine (central acting non-opiod analgesic) showed a significant improvement in ALT, AST and INR within 2 weeks compared to those who were not treated with NAC (Borlak et al., 2018). Furthermore, it is reported that NAC is benefit for tyrosine kinase inhibitors and norfloxacin-induced acute liver injury (Elliott et al., 2016; Patel et al., 2019). As shown in a prospective controlled study by an American ALF research group, which was conducted in 24 medical centers for 8 years in 173 patients with ALF caused by non-APAP agents, NAC could improve the survival rate of patients who were at the early stage of DILI-related ALF and had not undergone liver transplantation (Lee et al., 2009). Although the logistic regression analysis in their study did not show significant results, the NAC use also appeared to be associated with improved transplant free survival (Reuben, A. et al., 2010). However, a randomized, controlled treatment trial in children with ALF caused by non-APAP agents did not support a therapeutic role for NAC (Squires et al., 2013). Therefore, NAC is not recommended to treat children ALF caused by non-APAP agents, particularly pediatric patients of less than 2 years (Chalasani Naga et al., 2021). In a word, NAC was recommended for use in patients with early ALF, but was not recommended in children.

#### Glycyrrhizin Acid Preparation

Glycyrrhizin acid, extracted from the roots of the plant Glycyrrhiza glabra, is a triterpenoid compound, also known as glycyrrhizin for its sweet taste. It is hydrolyzed *in vivo* by glucuronidase to glucuronic acid and glycyrrhizin acid. At present, Glycyrrhizin acid preparation have been commonly used in the treatment of DILI in clinical application around the world, mainly including compound glycyrrhizin tablets, magnesium isoglycyrrhizinate (MgIG) and other drugs (Chen and Sun, 2011). It plays an anti-oxidation, anti-inflammation and hormone-like role in DILI treatment and protects liver against inflammatory damage. In China, Mao YM and coworkers carried out a multi-center, randomized, double-blind, multi-dose, positive drug parallel control clinical study of MgIG in the treatment of chronic liver disease (CLD) with elevated ALT among 412 patients including DILI. The results showed that MgIG can significantly reduce ALT and AST levels in patients, and the total effective rate was high (72.22%, 73.53%). Meanwhile, there was no obvious recurrence after withdrawal, and the incidence of adverse reactions was low (1.85%, 1.18%) (Mao et al., 2009). In 2015, MgIG has been approved by Chinese Food and Drug Administration (CFDA) to treat acute DILI, including the patterns of acute hepatocellular injury and mixed liver injury with significantly elevated serum ALT. Mao YM and coworkers then carried out a randomized, double-blind, multi-doses, active drug controlled, multi-center phase II trial involved 174 patients to assess the safety and efficacy of MgIG, as compared to tiopronin, a standard therapy for DILI in China and of which action was similar to NAC (Chiew et al., 2018). It was shown that both low-dose and high-dose MgIG lowered ALT level even at early stage of study drug administration. When compared with tiopronin (61.02%), the proportions of ALT normalization at week 4 were significantly greater in low dose MgIG (84.75%) and high dose MgIG (85.71%), respectively (Wang et al., 2019). Tang L et al. completed a phase III clinical trial of MgIG in the treatment of acute drug-induced liver injury induced by antineoplastic drugs. A total of 55 patients with antineoplastic DILI were treated with MgIG or tiopronin for 2 weeks respectively. The results revealed that after 1 week of treatment, the overall effective rate of MgIG group was significantly higher than that of the positive control group. After 2 weeks of treatment, the normalization rate of ALT and AST in MgIG group was significantly higher than that of tiopronin, but bilirubin levels were not mentioned. There was no statistical significance in the incidence of adverse reactions between the two groups (Tang et al., 2012).

#### Polyene Phosphatidylcholine

PPC is refined from phospholipids, which is extracted from soybeans. Accounting for about 52%, diacylphospholipidcholine (DLPC) is the main active ingredient. It can provide endogenous phospholipids to repair damaged liver cell and organelle membrane, so as to restore membrane function and increase the fluidity and stability of cell membrane, which protects from liver diseases caused by a variety of factors (Committee of the treatment with polyenphosphatidylcholine in patients with liver diseases, 2017). Lei X found that the ALT recurrence rate of PPC treatment in DILI was the same as MgIG (34.97%) through reanalysis of data from a multicenter retrospective cohort study in China (Lei et al., 2021). A single-center clinical study of 78 patients with anti-TB DILI found that PPC significantly increased serum heme oxygenase-1 (HO-1) and superoxide dismutase (SOD) levels and thus reduced oxidative stress responses (Liu et al., 2021). Moreover, PPC was often used as a control or combination agent in many clinical studies of DILI, demonstrating reliable efficacy in the treatment of DILI. Although only widely used in China so far, PPC has the potential clinical application value and is expected to be studied in more high-quality clinical studies in future.

#### Bicyclol

Bicyclol (4, 40-dimethoxy-5, 6, 50, 60-dimethylene-dioxy-2, 20-dicarboxylate biphenyl) is the first chemical new drug independently developed by China and used to treat inflammatory liver injury. It has been recommended by Russian physicians’ clinical guidelines for the treatment of DILI (Expert Committee on Clinical Application of Bicyclol, 2020). Its pharmacological activity mainly lies in its inhibition of the expression and activity of NF-κB, IL-1β, IL-18, TNF-α, TGF-β1 and other inflammatory regulatory factors induced by liver injury, as well as the production of reactive oxygen species (ROS) and nitric oxide (NO), reducing consumption of antioxidants such as GSH (Liu et al., 2017). Many clinical studies have shown that bicyclol demonstrated a good protective effect on the liver during acute and chronic liver injuries caused by a variety of chemical poisons, alcohols, and others agents (Expert Committee on Clinical Application of Bicyclol, 2020). Naiqiong W et al. conducted a multicenter and randomized controlled trial of 168 patients to explore the efficacy and safety of oral bicyclol versus polyene phosphatidylcholine in the treatment of statin-induced liver injury. After 2 and 4 weeks of treatment, the reduction of ALT in bicyclol (25 mg tid.po.) group was significantly higher than that of the polyene phosphatidylcholine (456 mg tid.po.) group, and there was no significant difference in the incidence of adverse reactions between the two groups (Naiqiong et al., 2017). Chen Y et al. evaluated the pharmacoeconomics of three therapeutic schemes in treating Anti-TB DILI of 225 newly treated TB patients. The results revealed that the efficacy of bicyclol in the treatment of Anti-TB DILI was superior to silybin and diammonium glycyrrhizinate, with good safety (Chen et al., 2018). In a meta-analysis involving 7 RCTs, it was also found that bicyclol had a good therapeutic effect on Anti-TB DILI, but all these 7 included studies were of low quality with high heterogeneity, and plausible publication bias was found (Wang et al., 2018). In addition, bicyclol had been reported to be effective for anti-neuropathy DILI (Li et al., 2018) and anti-psoriasis DILI (Zhou et al., 2015). More clinical studies are needed to provide sufficient evidence of its efficacy.

#### Silymarin

Silymarin is a flavonoid isolated for the first time in 1968 from the seed extract of milk thistle plant which is mainly a mixture of lignin-derived flavonols, including silybin, silydianin, silychristin, and isosilybin (Tittel and Wagner, 1977; Borah et al., 2013) with silybin is the most potent constituent (Committee of experts on silymarin therapy in patients with liver disease, 2016). Such drugs can maintain the fluidity of liver cell membrane through anti-lipid peroxidation and enhance the resistance of liver cell membrane to various injury factors, so as to protect against drug-induced liver injury (Chen and Sun, 2011). Enjalbert et al. (2002) reviewed the clinical data of 2,000 patients with liver injury caused by toadstool in 20 years and found that silymarin could significantly reduce the mortality rate of patients with toadstool poisoning (5.8%), while that of those who did not use silymarin was as high as 14.1%. There is also clinical evidence that early administration of silymarin can improve clinical outcome in children with toadstool poisoning (Grabhorn et al., 2013). Except treating liver injury caused by toxic substances, more importantly, there are also clinical studies indicating that it has the potential to treat DILI. Masoumeh Asgarshirazi et al. prospectively studied the efficacy and tolerance of Silymarin and Ursodeoxycholic acid (UDCA) in 46 anticonvulsive drugs induced hypertransaminasemia. The results showed that after 1 month of Silymarin treatment, ALT changes were significantly better than those in UDCA group, without obvious side effects (Masoumeh et al., 2015). A multicenter, retrospective, non-intervention cohort study conducted by Hong Zhao et al. revealed that Silybin alone improved liver function in patients with DILI (Zhao et al., 2021). However, at the same time, some studies have cast doubt on its therapeutic efficacy. A randomized double blind clinical trial enrolled 55 cases with anti-tuberculosis drugs induced liver injury were conducted in Iran (Majid. et al., 2019). The observational measures include severity of liver injury, the duration necessary for normalization of liver function, hospital stay and adverse effects. Consequently, there was not any statistically significant difference between silymarin and placebo groups. And the author concluded that silymarin is a safe herbal medication, but no effect to treat hepatic toxicity of anti-tuberculosis drugs.

### Anticholestatic Drugs

Multiple drugs have been reported to cause bile duct injury, which mostly affects the biliary epithelium of the interlobular ducts, thereby causing an inflammatory response directed at cholangiocytes. Cholestatic and mixed cholestatic and hepatocellular injury are two of the most severe manifestations of drug induced liver disease (DILD) (Bohan and Boyer, 2002). There is increasing evidence that drugs that are excreted by the liver into bile are prime candidates for producing cholestatic liver disease in the susceptible patient (Björnsson and Olsson, 2005). Many drugs target the biliary epithelium and result in “drug-induced cholangiopathy” and the “vanishing bile duct syndrome” (VBDS), characterized by loss of bile duct >50% due to inflammatory response and necrosis of the intralobular bile duct epithelium (Padda et al., 2011; Bessone et al., 2021). Most cases of drug induced cholestasis will resolve with withdrawal of the offending medication and not develop chronic liver disease. However, persistent cholestasis and hyperbilirubinemia lead to chronic cholestasis or cirrhosis, often suggesting a poor prognosis. In view of this, liver biopsy is particularly important for the differential diagnosis and treatment of drug-induced cholestasis (Bessone et al., 2019). So, the treatment of cholestasis or bile duct injury is particularly important in the management of DILI.

#### Ursodeoxycholic Acid

UDCA, a naturally occurring hydrophilic bile acid, which main mechanism is to reduce the cholesterol saturation index of bile and inhibit the absorption of cholesterol in intestine, has been reported to have a variety of hepatoprotective effect, such as cell protective, anti-apoptotic (Güldütuna et al., 1993), immunomodulatory (Calmus et al., 1990) and cholagogues effects (Beuers et al., 1993). It was the only FDA approved drug for treatment of primary biliary cholangitis (PBC) and has also been successfully used in various cholestatic liver diseases. Both European Association for the Study of the Liver (EASL) and Asia Pacific Association of Study of Liver (APASL) guidelines indicated that it was commonly used for DILI with cholestasis, although clinical evidence was not sufficient yet and therapeutic effect needs to be further studied (European Association for the Study of the Liver. 2019; Devarbhavi et al., 2021). A prospective pilot study investigated the hepatoprotective effectiveness of UDCA to tuberculosis- or non-tuberculosis mycobacterial-infected patients with DILI. The results revealed that all patients treated with UDCA show radiological and clinical improvement with no side effects (Lang et al., 2019). A retrospective study in China showed that UDCA can accelerate the recovery of TBIL, DBIL, Bile acid and GGT in patients with moderate and severe DILI while continuous treatment is required for more than 2 weeks (Sun et al., 2015). A clinical pilot study from Iran described pediatric patient with significant elevation (>2 ULN) in AST, ALT and γ-GGT) secondary to anticonvulsants confirmed at monthly follow up. The results showed a significant improvement of liver tests after treatment with UDCA for at least 24 weeks (*n* = 22) although due to uncontrolled design of this study a firm conclusion cannot be drawn (Asgarshirazi et al., 2015). However, some studies have refuted the effects of UDCA in DILI. Saito Z et al. retrospectively studied the effect of UDCA in sever DILI patients and found that UDCA did not improve normalization time or resolution rate compared with non-UDCA therapy. Further, the recovery time in UDCA treatment group was even longer (Saito et al., 2016). Additionally, UDCA has recently been recommended for drug-induced VBDS with cholestasis (Bessone et al., 2021), although a few cases of inefficacy have been reported (Xu et al., 2021). Recently, a systematic review of the role of UDCA in the treatment and prevention of DILI was performed, it commented that UDCA seems to have some benefits in the treatment of DILI. However, the design of the published studies does not allow a firm conclusion to be drawn on the efficacy of UDCA in DILI. A well designed RCT to evaluate the role of UDCA in DILI is needed. (Robles-Díaz Mercedes. et al., 2021).

#### S-Adenosylmethionine

SAMe is the active form of methionine in the body. It is formed from substrate L-methionine and ATP catalyzed by s-adenosylmethionine synthetase. Due to its transformation of methyl, sulfur and aminopropyl groups, SAMe is involved in the detoxification of bile acid metabolism and the generation of glutathione, improves cell membrane fluidity, promotes the synthesis and secretion of bile acid in many aspects, and prevents liver cell injury and necrosis (Finkelstein, 1990; Bottiglieri et al., 1994; Lieber, 1999). As early as 1990, SAMe was summarized as an effective treatment for drug-induced intrahepatic cholestasis, including estrogen, taurolithocholate, chlorpromazine, etc. (Almasio et al., 1990). It cound relieve the symptom of pruritus and restore serum total bilirubin and serum ALP towards normal. Perlamutrov Y et al. conducted an multicentric, non-interventional, prospective observational study enrolled 105 patients having DILI with cholestasis (by immunosuppressives) to characterize Russian population receiving SAMe as a hepatoprotectant against DILI triggered due to immunosuppressive drugs. The results revealed that the levels of ALT, AST, TBIL, ALP, and GGT significantly decreased after SAMe treatment and the symptoms of intrahepatic cholestasis such as pruritus, fatigue and jaundice improved (Perlamutrov et al., 2014). A prospective, multicenter, observational program found that SAMe significantly reduced ALT and AST in patient with chemotherapy induced liver injury after 2 weeks treatment (Noureddin et al., 2020). Santini D et al. observed 50 cancer patients who developed anticancer chemotherapy-induced liver toxicity, and received SAMe supplementation. They found that AST, ALT and LDH decrease after one or 2 weeks of SAMe treatment. This results clearly demonstrated a protective effect of SAMe in cancer chemotherapy-induced liver toxicity (Santini et al., 2003).

#### Cholestyramine

Cholestyramine, also known as Calleenamine, is a non-absorbable styrene anion exchange resin. After oral administration, it binds with intestinal cholic acid, hindering the reabsorption of cholic acid and increasing the excretion of cholic acid by 3∼15 times compared with normal. In clinical practice, cholestyramine was often used to relieve itchy skin symptoms in patients with cholestasis. As specific therapy, it was recommended by EASL and APASL guidelines that administration of cholestyramine may be used to decrease the course of hepatotoxicity induced by very selected drugs, such as leflunomide and terbinafine (European Association for the Study of the Liver. 2019; Devarbhavi et al., 2021). In the liver, leflunomide is a substrate of the hepatic microsomal enzymes CYP2C9, CYP3A4, and CYP2A1. Drugs that have an inducing or inhibitory effect on these hepatic microsomal enzymes carry the risk of adverse drug interactions with leflunomide (Alamri Raghad et al., 2021). Because the active metabolite of leflunomide is detectable in plasma until 2 years after discontinuation of the drug, cholestyramine must be given to enhance elimination from the body until plasma levels of leflunomide are undetectable (Østensen et al., 2006). Cholestyramine given orally in a dose of 8 g 3 times daily for 24 h reduces plasma concentrations of leflunomide by approximately 40% in 24 h and 65% in 48 h. A systematic review summarized a suggested algorithm for bile acid washout with cholestyramine for leflunomide toxicity, which can provide reference for clinical practice (Stine and Lewis, 2016).

### Immunosuppressants

Adaptive immune attack may be the final common event of DILI. Inflammatory responses are mainly a combination of immune activation and a series of related cellular and molecular events (Chalasani and Björnsson, 2010; Fontana, 2014). Intrahepatic inflammation caused by non-drug factors is an independent predisposing factor for DILI and also a factor that promotes the progression of DILI (Eisenberg-Lerner and Kimchi, 2012). Adaptive immune responses can mediate DILI and also lead to extrahepatic immune injury and then produce systemic manifestations including fever and rashes. Thus, immunosuppressants and anti-inflammatory drugs such as glucocorticoids (GCs) are also helpful in DILI treatment.

#### Glucocorticoids

As steroids, glucocorticoids (GCs) have been widely used in clinic practice, due to its anti-inflammatory, immunosuppressive, anti-allergic and anti-shock effects. GCs was recommended for severe DILI patients in cases where the patients’ serum TBIL levels are exacerbated under regular therapy, regardless of whether serum ALT level decreases or not (Hu and Xie, 2019). In addition, GCs could be empirically given to patients with marked signs of hypersensitivity or autoimmunity, and patients without remarkable improvement or even aggravation of biochemical indicators after withdrawal offending drug(s). A step-down therapy with gradually tapering off the dose of steroid seems to be used most often in clinical practice. Prednisone ranging from 15 to 20 mg/kg/day for 3 days was reported to treat successfully patients with severe DILI without obvious side effects. Abrupt withdrawal of the corticosteroids is not recommended because it may sometimes result in the rebound of liver injury (Hu and Xie, 2019). Analysis based on the American DILI Network Database showed that GCs were used in up to 82.0% of the DILI patients who died or underwent liver transplantation and 36.6% of DILI patients who survived (Fontana et al., 2014). Although the controversy about scientific evidence for efficacy of GCs has raged unabated for many years, some studies did confirm its effective role in DILI. Hou FQ and colleagues retrospectively investigated 70 patients DILI with hepatocellular injury, whose TBIL ≥10×ULN. The results revealed that the patients treated with GCs had higher DILI resolution rate with no side effects. Furthermore, the recovery time was longer in the patients who did no received GCs compared to those who did (Hou et al., 2012). A single-center retrospective study conducted by Hu PF et al. included 203 patients with severe DILI showed that 53 patients had a higher recovery rate and shorter recovery time after GCs treatment. They concluded that short-time use of corticosteroids is strongly recommended for severe DILI patients with hyperbilirubinemia (Hu et al., 2016). Sundaram S and Herroero-Herrero JI also respectively reported successful treatment of DILI with GCs (Herrero-Herrero and García-Aparicio, 2010; Sundaram et al., 2020). On the contrary, several investigations refuted the effects of GCs in DILI. Karkhanis J and coworkers retrospectively evaluated the effect of steroids in ALF. They collected clinical data of 131 patients with drug-induced ALF in which 16 received GCs treatment. The results revealed that GCs did not improve overall survival rate in drug-induced ALF (Karkhanis et al., 2014). Wan YM et al. enrolled 90 patients with severe DILI to investigate efficacy and safety of prednisone. The results revealed that prednisone therapy is safe but not beneficial, and even detrimental at a daily dose >40 mg for the treatment of severe DILI (Wan et al., 2019). Pang L and coworkers retrospectively studied the features and outcomes of hospitalized patients with DILI. They found that the recovery time and recovery rate of 32 acute DILI patients treated with GCs were not significantly improved compared with non-GCS group. However, encouragingly, the rate of resolution for patients with fulminant liver failure was superior to that of the non-steroid therapy group (57.1 vs. 25.0%) (Pang et al., 2018). These results suggest that GCs may have a potential therapeutic effect on DILI, but inappropriate dosage and timing may be counterproductive. Thus, GCs should be fully evaluated before application and used with great caution in DILI.

### Specific Drugs Treatment

In addition to hepatoprotective drugs, anticholestatic drugs, immunosuppressants, there are some specific drugs that play an important role in DILI treatment. For example, L-carnitine was used for liver injury caused by 3-keto-valproic acid (VPA). Anticoagulants such as low-molecular-weight heparins and defibrotide were used for sinusoidal obstruction syndrome (SOS)/hepatic veno-occlusive disease (VOD).

#### L-Carnitine

LC is a vitamin-like amino acid derivative, mainly derived from daily diet, especially red meat and dairy products. It plays a key role in physiological metabolism include promoting the transport of long-chain fatty acids, preventing the accumulation of acetyl-CoA in mitochondria, and thus promoting the β oxidation of fatty acids, which further are widely used in weight-loss products (Lheureux et al., 2005). In 1982, reduced serum free carnitine as well as reduced levels of 3-keto-valproic acid (VPA), the main metabolite of β-oxidation of VPA, was first reported by Bohles and coworkers in a 3-year-old girl who developed acute liver disease with typical features of Reye’s syndrome after treatment with VPA for 6 months (Böhles et al., 1982). Subsequently, Krähenbühl et al. (1995) found that the LC content of plasma and liver were reduced in a patient with fatal liver injury caused by VAP. Bohan TP and coworkers conducted a retrospective cohort study of 92 patients with VAP-induced fatal hepatotoxicity. They found that 48% survived of 42 patients treated with LC and of 50 patients who only received supportive treatment, 10% survived (Bohan et al., 2001). Although low quality grading of evidence, EASL and APASL guidelines both recommend LC for liver injury caused by VPA.

#### Anticoagulants

Drug-induced liver vascular injury is relatively rare with unknown pathogenesis, in which the target cells can be hepatic sinus endothelial cells, hepatic venules, as well as the hepatic vein and the portal vein, and the clinical types include sinusoidal obstruction syndrome (SOS)/hepatic veno-occlusive disease (VOD) (Gao et al., 2012; Fan and Crawford, 2014), peliosis hepatis (PH) (Yu et al., 2014; Kootte et al., 2015), Budd-Chiari syndrome (BCS), idiopathic portal hypertension (IPH) induced by portal sclerosis, etc. Drugs that caused vascular liver injury usually included some chemotherapeutic agents such as Irinotecan or Oxaliplatin (Bai et al., 2016), herbs containing pyrrolizidine alkaloids (Leise et al., 2014), etc. There were also clinical evidences that oral contraceptives (OC) use played a role to enhance the underlying thrombophilia, due the production of clotting factor (Scarsi et al., 2016), leading the development of hepatic vein thrombosis (Perarnau and Bacq, 2008). For SOS/VOD, supportive symptomatic treatment is the basic pyrrolidine alkaloid-related hepatic sinusoidal obstruction syndrome (PA-HSOS) treatment regimen. And the early use of anticoagulants such as low-molecular-weight heparins after ruling out contraindications may have some therapeutic effect. Wang et al. summarized the long-term follow-up results of different PA-HSOS treatment regimens in a retrospective monocenter study. 85 patients with Gynura segetum caused VOD were divided into two groups according to whether they had received anticoagulant therapy (low molecular weight heparin, warfarin) on the basis of treatment of liver function protection and microcirculation improvement. Among 22 patients who received no anticoagulant treatment, 6 (27.3%) patients were cured and 14 (63.6%) patients died during the treatment period. Among 63 patients treated with anticoagulant, 6 (9.5%) patients died and 36 (57.1%) patients were cured. The results showed that the cure rate was higher than that of non-anticoagulant treatment (Wang et al., 2016). Additionally, defibrotide is the only drug demonstrated to be effective for the prevention and treatment of hematopoietic stem cell transplantation related hepatic sinusoidal obstruction syndrome (HSCT–HSOS) (Richardson et al., 1998; Dignan et al., 2013; Richardson et al., 2016).

### Combination of Therapeutic Drugs

The mixed phenotype of DILI was often associated with multiple manifestations of cholestasis and hepatocyte injury, with significantly increased all TBIL, ALP, ALT and AST. Despite the lack of adequate evidence-based medical evidence, it is not uncommon for clinicians to choose two even more hepatoprotective drugs for DILI treatment in clinical practice. A clinical study involving 40 Chinese patients with DILI during chemotherapy after radical gastritis showed that PPC combined with bicyclol significantly reduced serum ALT, AST and ALP levels compared with PPC treatment (Wu et al., 2020). A clinical study conducted by Wang HX et al. found that MgIG combined with NAC treatment significantly reduced serum malondialdehyde (MDA), interleukin-6 (IL-6) and procalcitonin (PCT), while increased the levels of glutathione peroxidase (GSH-Px) and superoxide dismutase (SOD) in DILI patients, suggesting a good anti-inflammatory and antioxidant effect (Wang et al., 2020). Qing Xie’s team retrospectively analyzed the efficacy of UDCA combined with SAMe in treatment of cholestatic DILI, and found that patients received the combination therapy had a better efficacy rate than SAMe alone, with better safety (Ye et al., 2017). In a comparative analysis of clinical data from 15 patients with severe DILI, Wree A et al. found that Steroid combined with UDCA therapy appeared to be safe, and leads to a more rapid reduction in bilirubin and transaminases (Wree et al., 2011). The combination of two or more anti-inflammatory and hepatoprotective drugs has not been recommended in DILI guidelines circulated internationally, and it is urgent to carry out multi-center clinical studies with large samples to provide more adequate evidence-based medical evidence (Yue et al., 2017).

## Non-Pharmacological Support Therapy

Most cases of DILI are self-limiting and resolve with prompt identification and cessation of the offending agent. The patients with abnormal liver function after drug withdrawal and chronic DILI (persistent abnormalities in liver-associated enzymes at 3–6 months after drug withdrawal) (Andrade et al., 2006; Chalasani et al., 2015) are often require pharmacotherapy as described above. However, for DILI patients with severe hepatic encephalopathy, coagulation disorders or decompensated liver cirrhosis, non-pharmacological support therapy need to be considered, such as hemodialysis, peritoneal dialysis, artificial extracorporeal liver support and liver transplantation.

### Liver Transplantation

Acute DILI had been reported to occur in 5–10% of patients hospitalized for jaundice (Björnsson et al., 2003), and more often evolved to fulminant hepatic failure than other causes of acute hepatic injury in Western countries (Ostapowicz et al., 2002; Wei et al., 2007). DILI is an important cause of ALF with significant morbidity and mortality. According to Russo MW et al. statistical analysis of the United Network for Organ Sharing (UNOS) liver transplant database from 1990 to 2002, it showed that liver transplantation for drug hepatotoxicity accounted for 15% of liver transplants for ALF in US, in which mostly attributed to APAP (Russo et al., 2004). Thus, Liver transplantation should be considered for patients with ALF/SALF who present with hepatic encephalopathy and severe coagulation disorders, as well as decompensated cirrhosis (Reuben et al., 2010). All the American College of Gastroenterology (ACG), EASL and APASL clinical practice guidelines pointed out, liver transplantation was still the primary rescue treatment for ALF (European Association for the Study of the Liver. 2019; Chalasani Naga et al., 2021; Devarbhavi et al., 2021), with 1 and 5 years survival benefits of about 70–80% in liver transplants for DILI-ALF (Adam et al., 2018).

### Artificial Extracorporeal Liver Support

By means of *in vitro* mechanical, chemical or biological devices, AELS is a temporary or partial replacement of liver function to assist in the treatment of liver dysfunction or related diseases, mainly including biological artificial liver (BAL), no-biological artificial liver (NBAL) and their mixture. NBAL plays an important role in clinical practice, but its curative effect remains to be discussed. An open randomized controlled trial in regard to High-volume plasma exchange (HVP) in patients with ALF showed that treatment with HVP can increase liver transplant-free survival (58.7 vs. 47.8%), and the incidence of severe adverse events was similar to control group (Larsen et al., 2016). But, another trial about extracorporeal albumin dialysis with the molecular adsorbent recirculating system in acute-on-chronic liver failure revealed that the beneficial effect of it cannot be verified (Bañares et al., 2013). A review also concluded that the sole use of devices such as hemodialysis and albumin dialysis aimed to remove toxins is not sufficient to improve survival in patients with ALF (Larsen, 2019).

## Conclusion and Future Perspectives

DILI is one of the most common and serious adverse drug reactions (ADRs), leading to acute liver failure (ALF) and even death in severe cases (Li et al., 2007; Miguel et al., 2012; Björnsson et al., 2013). Up to now, there is still lack of simple, objective and specific diagnostic indexes and treatment methods due to its complex pathogenesis. Thus, it is vital to identify the offending drug(s) and prompt withdrawal before irreversible liver damage. The DILI Collaboration Network (DILIN) was founded in the United States in 2003, and a prospective study of DILIN (DILIN-PS) was initiated next year (Fontana et al., 2009). The LiverTox website (http://www.livertox.nih.gov) set up by DILIN in 2012 and HepaTox website (http://www.hepatox.org) by Chinese Medical Association drug-induced liver disease Group in 2014 records information on more than 1,000 common drugs for liver damage. It can provide a basis for avoiding the use of potential hepatotoxic drugs in clinical practice.

For pharmacological therapy of DILI, there is a lack of normative guidance or guidelines. There is usually a complete or near complete resolution of DILI within a matter of days to weeks after offending drug withdrawal. The starting point of protective drugs intervention is still difficult to choose. Therefore, dynamic monitoring of liver biochemical parameters is recommended by all clinical practice guidelines. Based on the type of target cells damaged, patients tend to show different clinical phenotypes of DILI with a distinction in liver biochemical indicators. In this paper, we reviewed a large amount of evidence-based medical studies and summarized the commonly used drugs for DILI in clinical practice. NAC, MgIG, PPC, bicyclol, silymarin are potential hepatoprotective drugs for DILI. In anticholestatic drugs, UDCA, SAMe, cholestyramine showed therapeutic value for DILI. LC is recommended for liver injury caused by VPA. GCs is suitable for patients with obvious hypersensitivity or autoimmune symptoms and no obvious improvement in liver biochemical indicators after withdrawal. Anticoagulants, such as low-molecular-weight heparins and defibrotide, play irreplaceable roles in some special types of DILI (eg. PA-HSOS, etc.). They could provide reference for the selection and combined application of protective drugs for different clinical phenotypes of DILI. Nevertheless, the definite therapeutic effects of these drugs are still to be confirmed by prospective randomized and controlled studies.

The onset of DILI is a complex process, and its pathogenesis has become a research hotspot in recent years. With further research into the pathological mechanisms and the rapid development of techniques, many new therapeutic drugs may shed light on DILI clinical management. Emerging therapeutic interventions for DILI include gene therapy, nano-therapy, structural modification of toxic drugs, etc. For example, the role of C-X-C motif chemokine ligand-9 (CXCL9) in APAP-induced liver injury was reported crucial recently. Despite the lack of clinical trials, hepatocyte apoptosis of APAP-treated mice decreased following administration of a CXCL9 neutralizing antibody or CXCR3 gene knockout (Song et al., 2019). In humans, genetically inherited defects in BSEP expression or activity cause cholestatic liver injury, and many drugs that cause cholestatic drug-induced liver injury (DILI) in humans have been shown to inhibit BSEP activity *in vitro* and *in vivo* (Saran et al., 2021). Attempts at molecular structural modification of hepatotoxic drugs that inhibit BSEP may provide new strategies for treatment of cholestatic DILI (Warner et al., 2012). Exciting work in the field of nanotechnology holds the promise of developing novel treatment and prevention approaches for DILI in the not-too-distant future. The ability to deliver inhibitors of inflammatory cytokines, apoptosis and other cellular events leading to liver cell necrosis and death directly to hepatocytes or other substructures to prevent DILI is an attractive possibility for the future, and is being actively pursued (Isoda et al., 2011; Reddy and Couvreur, 2011; Cui et al., 2012; Momen-Heravi et al., 2014).
